# Photobiomodulation Mitigates Cerebrovascular Leakage Induced by the Parkinsonian Neurotoxin MPTP

**DOI:** 10.3390/biom9100564

**Published:** 2019-10-04

**Authors:** Mia San Miguel, Kristy L. Martin, Jonathan Stone, Daniel M. Johnstone

**Affiliations:** Discipline of Physiology and Bosch Institute, University of Sydney, Sydney, NSW 2006, Australia; smmia27@gmail.com (M.S.M.); kristy.martin@sydney.edu.au (K.L.M.); jonathan.stone@sydney.edu.au (J.S.)

**Keywords:** photobiomodulation, MPTP, Parkinson’s disease, cerebrovasculature, mouse

## Abstract

The neurotoxin 1-methyl-4-phenyl-1,2,3,6-tetrahydropyridine (MPTP) is commonly used to model Parkinson’s disease (PD) as it specifically damages the nigrostriatal dopaminergic pathway. Recent studies in mice have, however, provided evidence that MPTP also compromises the integrity of the brain’s vasculature. Photobiomodulation (PBM), the irradiation of tissue with low-intensity red light, mitigates MPTP-induced loss of dopaminergic neurons in the midbrain, but whether PBM also mitigates MPTP-induced damage to the cerebrovasculature has not been investigated. This study aimed to characterize the time course of cerebrovascular disruption following MPTP exposure and to determine whether PBM can mitigate this disruption. Young adult male C57BL/6 mice were injected with 80 mg/kg MPTP or isotonic saline and perfused with fluorescein isothiocyanate FITC-labelled albumin at various time points post-injection. By 7 days post-injection, there was substantial and significant leakage of FITC-labelled albumin into both the substantia nigra pars compacta (SNc; *p* < 0.0001) and the caudate-putamen complex (CPu; *p* ≤ 0.0003); this leakage partly subsided by 14 days post-injection. Mice that were injected with MPTP and treated with daily transcranial PBM (670 nm, 50 mW/cm^2^, 3 min/day), commencing 24 h after MPTP injection, showed significantly less leakage of FITC-labelled albumin in both the SNc (*p* < 0.0001) and CPu (*p* = 0.0003) than sham-treated MPTP mice, with levels of leakage that were not significantly different from saline-injected controls. In summary, this study confirms that MPTP damages the brain’s vasculature, delineates the time course of leakage induced by MPTP out to 14 days post-injection, and provides the first direct evidence that PBM can mitigate this leakage. These findings provide new understanding of the use of the MPTP mouse model as an experimental tool and highlight the potential of PBM as a therapeutic tool for reducing vascular dysfunction in neurological conditions.

## 1. Introduction

1-methyl-4-phenyl-1,2,3,6-tetrahydropyridine (MPTP) is a neurotoxin commonly used to model Parkinson’s disease (PD). As a highly lipophilic molecule, MPTP readily crosses the blood-brain barrier (BBB), whereupon it is converted into 1-methyl-4-phenylpyridinium (MPP^+^) by monoamine oxidase B in astrocytes; MPP^+^ enters dopaminergic neurons through the dopamine transporter, where it inhibits complex I of the mitochondrial electron transport chain, preventing adenosine triphosphate (ATP) synthesis and ultimately causing cell death [1]. Given the ability of MPTP to selectively and rapidly destroy dopaminergic neurons in the nigrostriatal pathway and reproduce some of the functional deficits associated with PD [2,3,4,5], it has been utilized as a PD mimetic in a variety of model species in thousands of studies.

Recently, studies of mice have provided evidence that exposure to MPTP can also compromise the integrity of the brain vasculature [6,7,8,9,10]. Most of these studies used a fluorescent tracer, such as fluorescein isothiocyanate (FITC)-labelled albumin or FITC-dextran injected into the circulation, and assessed the nigrostriatal region for the leakage of albumin/dextran (normally too large a molecule to pass across the BBB) into the neuropil. A limitation of studies to date is that all have been cross-sectional, assessing just a single time point post-injection (usually 3 or 7 days) [6,7,8,9,10]. One aim of the present study was to assess multiple time points in order to define the timeline of MPTP-induced damage to the vasculature of the substantia nigra pars compacta (SNc) and caudate-putamen complex (CPu).

MPTP models have been employed extensively for trialing therapeutic interventions for PD. For example, we have utilized MPTP-injected mice to assess the neuroprotective efficacy of photobiomodulation (PBM), the irradiation of tissue with low-intensity red or near-infrared light. In mice administered MPTP following sub-chronic or chronic injection schedules, transcranial PBM significantly mitigates the loss of dopaminergic cells in the SNc [11,12,13,14,15,16,17,18], astrogliosis in the SNc and CPu [11] and locomotor deficits as assessed by the open field test [13,15,16,17]. Furthermore, light irradiation of the body (remote PBM) but not the head significantly mitigates the loss of SNc dopaminergic cells in mice exposed to 50 mg/kg MPTP [12,19,20]. 

Despite the extensive evidence that PBM can protect against MPTP-induced neuronal loss, gliosis and functional deficits, its effects on the cerebrovasculature have not yet been explored. Thus, a second aim of this study was to determine whether transcranial PBM can mitigate MPTP-induced cerebrovascular leakage, at the time point when greatest damage is observed.

## 2. Materials and Methods 

### 2.1. Animals

All protocols were approved by the University of Sydney Animal Ethics Committee (Project Number 2017/1128, modification approved 20 September 2018). All experiments used male C57BL/6 mice, aged 12 weeks. Mouse weights ranged between 24 g and 30 g. Separate cohorts of mice were used for addressing the two aims of the study: the first cohort contained 28 mice and the second cohort contained 20 mice.

Mice in the first cohort were randomly allocated to one of 8 experimental groups, corresponding to a specific time point post-injection at which they were sacrificed. Mice injected with MPTP were sacrificed at either 1 day (*n* = 4), 2 days (*n* = 4), 3 days (*n* = 4), 7 days (*n* = 4) or 14 days (*n* = 4) post-injection, while control mice injected with saline were sacrificed at either 3 days (*n* = 4), 7 days (*n* = 2) or 14 days (*n* = 2) post-injection. 

Mice in the second cohort were randomly allocated one of three different experimental groups: (i) saline injection, sham treatment (*n* = 8), (ii) MPTP injection, sham treatment (*n* = 6) and (iii) MPTP injection, PBM treatment (*n* = 6). Mice in this cohort were sacrificed at 7 days post-injection. 

### 2.2. MPTP and Saline Injections

Mice were randomly allocated to receive intraperitoneal injections of either the parkinsonian neurotoxin MPTP (dissolved in isotonic saline) or vehicle (isotonic saline). Mice in the MPTP groups received four injections of 20 mg/kg (total dose of 80 mg/kg), with a period of 2 h between each injection [21]. Mice in the saline control groups received four injections of an equivalent volume of isotonic saline. 

### 2.3. PBM Treatment

Prior to PBM treatment, the fur was shaved from the head of mice using clippers, to increase the penetration of light to the brain. Photobiomodulation (670 nm, 50 mW/cm^2^) was applied to the shaved head of the mouse using a WARP10 LED panel (Quantum Devices Inc, Barneveld, WI, USA), for 3 min per day over 7 days, commencing 24 h following MPTP injections. Mice were restrained by lightly scruffing the fur behind the neck; mice in the sham group were similarly restrained but light treatment was not applied.

### 2.4. Animal Perfusion, Tissue Collection and Preparation

Mice were anesthetized with 50 mg/kg sodium pentobarbitone and dissected to expose the heart and pleural cavity. Heparin (3 U in 100 µL PBS) was injected directly into the left ventricle of the heart. Fluorescein isothiocyanate-labelled albumin (FITC-LA), prepared in PBS at a concentration of 5 mg/mL, was perfused through the left ventricle at a flow rate of 1.5 mL/min using a 23G butterfly intravenous perfusion kit connected to a 30 mL syringe, dispensed by a syringe auto pump injector. Each mouse was perfused with a total of 5 mL of FITC-LA solution.

Following perfusion, brains were removed and immersion-fixed in 10% formalin for 24 h in light-protected containers, followed by cryoprotection in 30% sucrose in PBS for 3 days. Following cryoprotection, each brain was trimmed in the coronal plane at the level of the superior colliculus and the cerebrum was embedded and frozen in TissueTek OCT compound. Embedded brains were cryosectioned in the coronal plane using a Leica Cryostat; the entire SNc (Bregma −3.88 mm to −2.82 mm) was sectioned as a 1:3 series at a thickness of 30 µm, while the CPu (Bregma 0.26 to 1.54) was sectioned as a 1:3 series at a thickness of 50 µm. Sections were collected onto poly-l-lysine/gelatin coated slides and stored at –20 °C until required.

### 2.5. Imaging and Analysis of FITC-LA Distribution

For imaging of FITC-LA, slides containing one series of sections from the SNc and CPu were placed in an oven at 37 °C for 1 h, followed by removal of OCT with 70% ethanol (5 min). Sections were mounted with anti-fade medium (4% propyl gallate, 90% glycerol in PBS), coverslipped, sealed and stored at –20 °C.

Sections were imaged using the 20x objective of a Zeiss LSM 800 confocal microscope (Zeiss, Oberkochen, Germany), with the operator blinded to sample identity. The green FITC (488 nm) channel was used to generate images of vessels and leakage in the SNc and the CPu. As the SNc has a wing-like shape, a 3 × 2 tile scan was performed in conjunction with a z-stack (20–24 frames) to image the entire region (~400 µm × 250 µm). Images were collected from 2–3 SNc sections per animal, with sections matched based on anatomical landmarks, such as mamillary body size and ventricle size. As the CPu represents a much larger region, a z-stack of a representative field of view was performed without a tile scan (30–34 frames). Images were collected from 4 CPu sections per animal.

**Figure 1:**
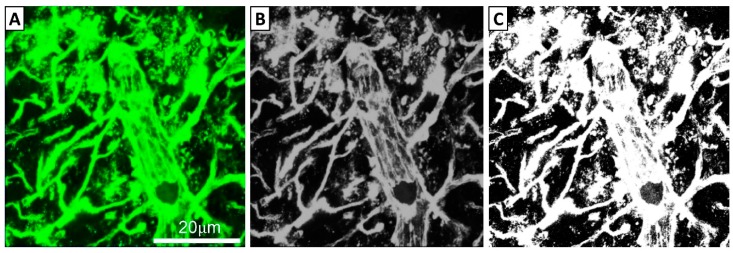
Process of image transformation prior to quantification. (**A**) Fluorescent images obtained from a Zeiss LSM800 Confocal Microscope were converted to (**B**) 8-bit grayscale images using ImageJ software. (**C**) Images were then thresholded with a preset value of 50 using ImageJ.

Images were compressed to show the entire tile scan/z-stack in a single image. For both the SNc and CPu, colour images were converted to 8-bit grayscale images in ImageJ, and a consistent threshold of 50 was applied to all grayscale images to generate black and white images (Figure 1). The total area comprising white (i.e., above threshold) pixels was calculated as a proportion of the total sampled area.

Statistical analyses were performed using GraphPad Prism 7.02 (GraphPad Software, San Diego, CA, USA). Outlier testing used the ROUT method (Q = 1%). One-way analysis of variance (ANOVA) was used to compare the means of different experimental groups; where significance (*p* < 0.05) was indicated, Tukey’s multiple comparisons test was used for pairwise comparisons.

## 3. Results

### 3.1. Time Course of MPTP-Induced Dysfunction of the Cerebrovasculature

#### 3.1.1. Perfusion Time Point Had No Obvious Effect on the Cerebrovasculature of Saline-Injected Controls

Control mice injected with isotonic saline and allowed to survive for either 3, 7 or 14 days showed no noticeable vascular leakage. This was supported by quantitative analysis, which detected no difference in FITC-LA labelling across the three time points (data not shown). In the absence of an effect of the time point, all saline-injected animals were combined into a single experimental group for subsequent analyses.

#### 3.1.2. MPTP-Induced Cerebrovascular Leakage in the SNc Peaks at 7 Days Post-Injection

**Figure 2:**
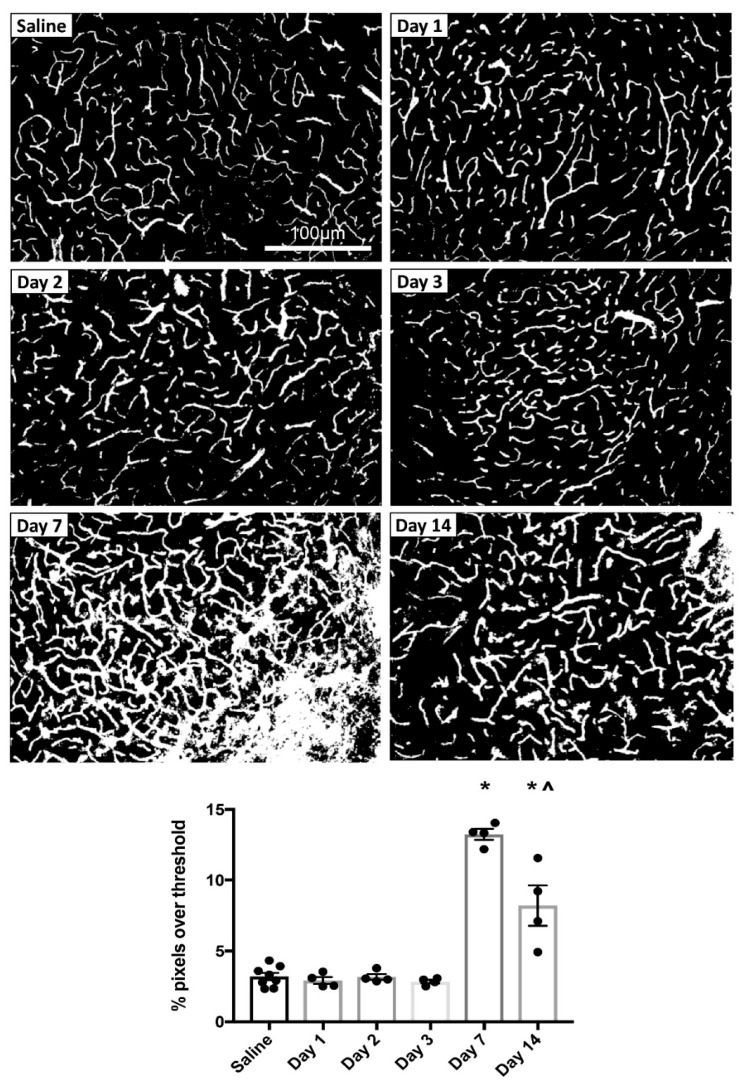
MPTP-induced vascular leakage in the SNc. Top panels: Representative thresholded images of FITC-LA labelling in the SNc of mice injected with MPTP and perfused at 1, 2, 3, 7 or 14 days post-injection, compared to saline-injected control (top left). Scale bar in the top left image applies to all images. Bottom panel: Quantification of the percentage of pixels above threshold, as determined from thresholded FITC-LA images. Data are presented as mean ± SEM (*n* = 8 for saline, *n* = 4 for all other time points). * *p* < 0.0001 vs. Saline, Day 1, Day 2 and Day 3, ^ *p* < 0.0001 vs. Day 7.

To explore the time course of MPTP-induced changes in cerebrovascular permeability, mice were injected with MPTP and allowed to survive for either 1, 2, 3, 7 or 14 days before perfusion with FITC-LA. Representative thresholded images of FITC-LA in the SNc of mice at each time point are shown in Figure 2. Qualitative analysis suggested no leakage at earlier time points (days 1–3); most sections were indistinguishable from saline-injected controls. However, at day 7 there was extensive leakage of FITC-LA beyond the vessel walls and into the neuropil. Some leakage was also apparent at the day 14 time point but did not appear as extensive as at day 7.

Quantification of FITC-LA in SNc sections from mice perfused at different time points is presented in Figure 2. Relative to saline-injected controls, there was no difference in the extent of FITC-LA labelling for mice perfused within 3 days of MPTP injection. However, mice perfused 7 days after MPTP injection showed a > 4-fold increase in FITC-LA labelling, while mice perfused 14 days after MPTP injection showed a > 2-fold increase in FITC-LA labelling, relative to saline-injected controls. One-way ANOVA revealed a significant difference in group means (F(5, 22) = 54.46, *p* < 0.0001), with post-hoc Tukey’s multiple comparisons test revealing significant differences at 7 days (*p* < 0.0001) and 14 days (*p* < 0.0001) post-injection relative to all other time points. In addition, there was a significant difference in FITC-LA labelling between the 7 and 14 day time points (*p* < 0.0001), suggesting that MPTP-induced cerebrovascular leakage in the SNc peaks at 7 days post-injection.

#### 3.1.3. MPTP-Induced Cerebrovascular Leakage in the CPu Peaks at 7 Days Post-Injection

**Figure 3:**
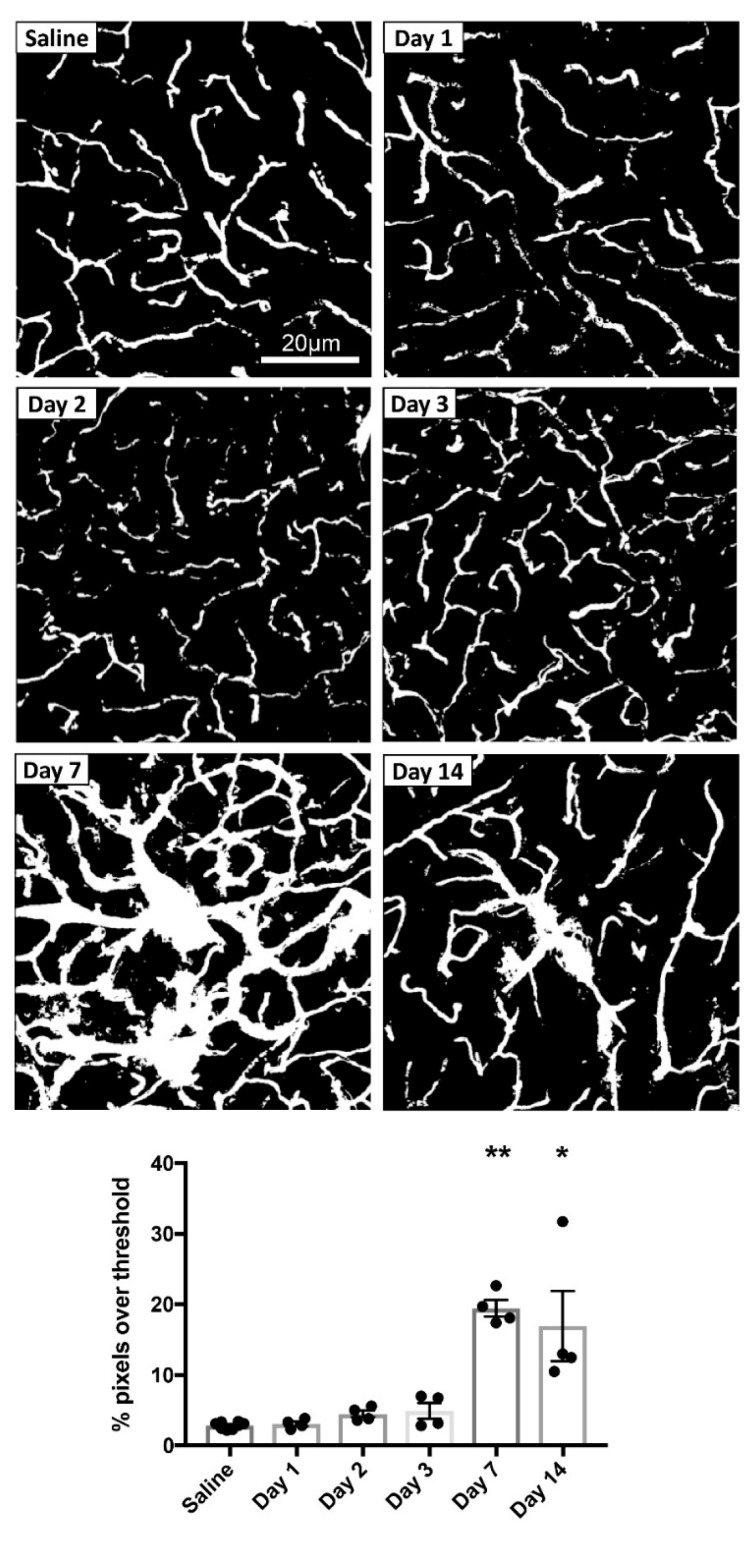
MPTP-induced vascular leakage in the CPu. Top panels: Representative thresholded images of FITC-LA labelling in the CPu of mice injected with MPTP and perfused at 1, 2, 3, 7 or 14 days post-injection, compared to saline-injected control (top left). Scale bar in the top left image applies to all images. Bottom panel: Quantification of the percentage of pixels above threshold, as determined from thresholded FITC-LA images. Data are presented as mean ± SEM (*n* = 8 for saline, *n* = 4 for all other time points). ** *p* ≤ 0.0003 vs. Saline, Day 1, Day 2 and Day 3, * *p* ≤ 0.003 vs. Saline, Day 1, Day 2 and Day 3.

In addition to assessing the SNc, a similar analysis of FITC-LA distribution was also conducted for the CPu. Representative thresholded images of FITC-LA in the CPu of mice at each time point are shown in Figure 3. As in the SNc, there was no apparent leakage of FITC-LA in the CPu at days 1-3. However, by day 7, there was substantial leakage of FITC-LA beyond the vessel walls, with the day 14 time point also showing a moderate amount of leakage.

Quantification of FITC-LA in CPu sections from mice perfused at different time points is presented in Figure 3. Relative to saline-injected controls, there was no difference in the extent of FITC-LA labelling for mice perfused within 3 days of MPTP injection. However, mice perfused 7 days after MPTP injection showed an almost 7-fold increase in FITC-LA labelling, while mice perfused 14 days after MPTP injection showed a 6-fold increase, relative to saline-injected controls. One value in the Day 14 group deviated considerably from the other three but was not identified as an outlier using the ROUT method and was therefore retained in the analysis. One-way ANOVA revealed a significant difference in group means (F(5, 22) = 16.38, *p* < 0.0001), with post-hoc Tukey’s multiple comparisons test revealing significant differences at 7 days (*p* ≤ 0.0003) and 14 days (*p* ≤ 0.003) post-injection relative to all other time points. Unlike for the SNc, there was no significant difference between the 7 and 14 day time points (*p* = 0.94).

#### 3.1.4. FITC-LA Labelling in the SNc and CPu are Strongly Correlated

**Figure 4:**
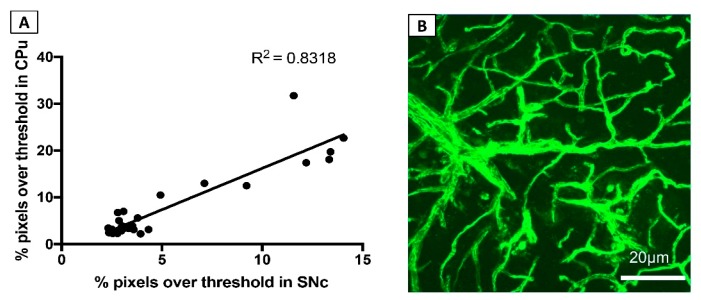
MPTP induces widespread cerebrovascular dysfunction. (**A**) Correlation of FITC-LA labelling in CPu and SNc. (**B**) Representative image of FITC-LA labelling in the neocortex of mice at 7 days post-injection.

To investigate the consistency of MPTP-induced cerebrovascular leakage across two key regions of the brain—the SNc and CPu—a linear regression analysis was performed. This analysis revealed a strong correlation between FITC-LA labelling in the SNc and CPu, returning an *r^2^* value of 0.83 (*p* < 0.0001) (Figure 4A). 

#### 3.1.5. MPTP-Induced Cerebrovascular Leakage is Not Confined to the SNc and CPu

In terms of neuronal damage and death, the destructive effects of MPTP are mostly confined to the nigrostriatal dopaminergic pathway, with no obvious neurodegeneration observed in other regions of the brain [5]. However, it is unknown whether the effects of MPTP on the brain vasculature are similarly confined or more widespread.

To explore this possibility, a qualitative analysis of FITC-LA labelling in other brain regions, such as the neocortex, was undertaken. Most animals that had been administered MPTP appeared to have extensive leakage in areas such as the neocortex at day 7 and day 14 post-injection (Figure 4B). The observation that the damaging effects of MPTP on the vasculature are not confined to brain regions showing obvious neurodegeneration suggests that MPTP-induced cerebrovascular dysfunction is not solely a consequence of MPTP-induced neuronal death.

### 3.2. Effects of Transcranial PBM on MPTP-Induced Dysfunction of the Cerebrovasculature

The results above indicate that, of the time points tested, cerebrovascular leakage is greatest at 7 days post-MPTP injection. Thus, 7 days was selected as the survival period for experiments investigating whether transcranial PBM mitigates MPTP-induced cerebrovascular dysfunction.

#### 3.2.1. Transcranial PBM Mitigates MPTP-Induced Cerebrovascular Leakage in the SNc

**Figure 5:**
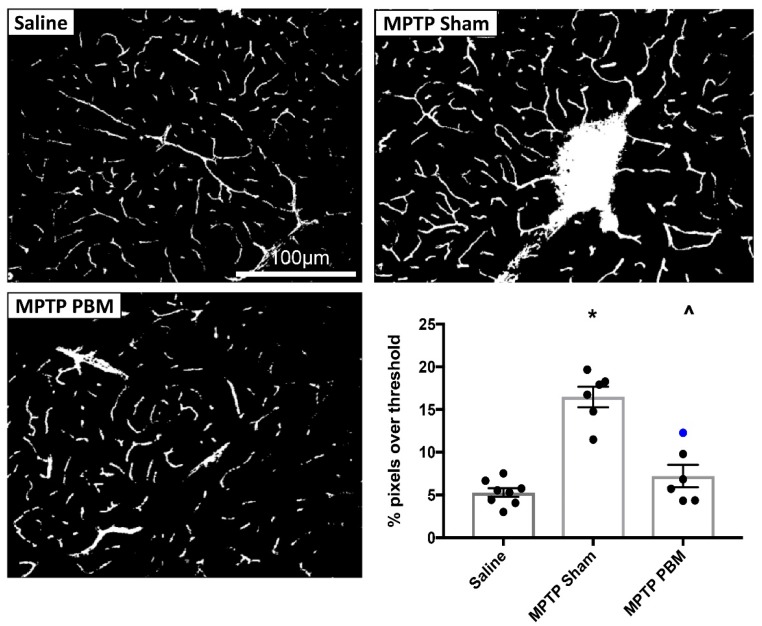
Transcranial PBM mitigates MPTP-induced vascular leakage in the SNc. Top and bottom left panels: Representative thresholded images of FITC-LA labelling in the SNc of mice injected with MPTP and exposed to daily PBM treatment (MPTP PBM) or sham treatment (MPTP Sham), as well as saline-injected controls (Saline). Scale bar in the top left image applies to all images. Bottom right panel: Quantification of the percentage of pixels above threshold, as determined from thresholded FITC-LA images. Data are presented as mean ± SEM (*n* = 8 for Saline, *n* = 6 for MPTP Sham, *n* = 6 for MPTP PBM). * *p* < 0.0001 vs. Saline; ^ *p* < 0.0001 vs MPTP Sham. Blue dot represents the unshaved mouse.

Mice were randomized to three experimental groups: (i) saline injections, sham treatments, (ii) MPTP injections, sham treatments and (iii) MPTP injections, PBM treatments. Representative thresholded images of the SNc of mice from each experimental group are shown in Figure 5. Qualitatively, inspection of the images from each animal generally showed extensive FITC-LA leakage in the MPTP-sham group, while the MPTP-PBM group showed minimal leakage, with some cases being indistinguishable from saline-injected control animals.

Quantification of FITC-LA labelling in SNc sections is presented in Figure 5, with the blue marker representing a PBM-treated MPTP mouse that was not shaved, and thus likely to have received less light energy to the brain. Relative to saline-injected controls, mice in the MPTP-sham group showed a >3-fold increase in FITC-LA labelling. Mice in the MPTP-PBM group showed similar values to the saline-injected controls, with the unshaved animal returning the highest value (blue marker). One-way ANOVA revealed a significant difference in group means (F(2, 17) = 36.14, *p* < 0.0001), with post-hoc Tukey’s multiple comparisons test revealing significant differences (*p* < 0.0001) between the MPTP-sham group and both the MPTP-PBM group and saline-injected controls. There was no significant difference between the saline control and MPTP-PBM groups (*p* = 0.36), confirming the qualitative impression that transcranial PBM strongly mitigates MPTP-induced cerebrovascular leakage in the SNc.

#### 3.2.2. Transcranial PBM Mitigates MPTP-Induced Cerebrovascular Leakage in the CPu

**Figure 6:**
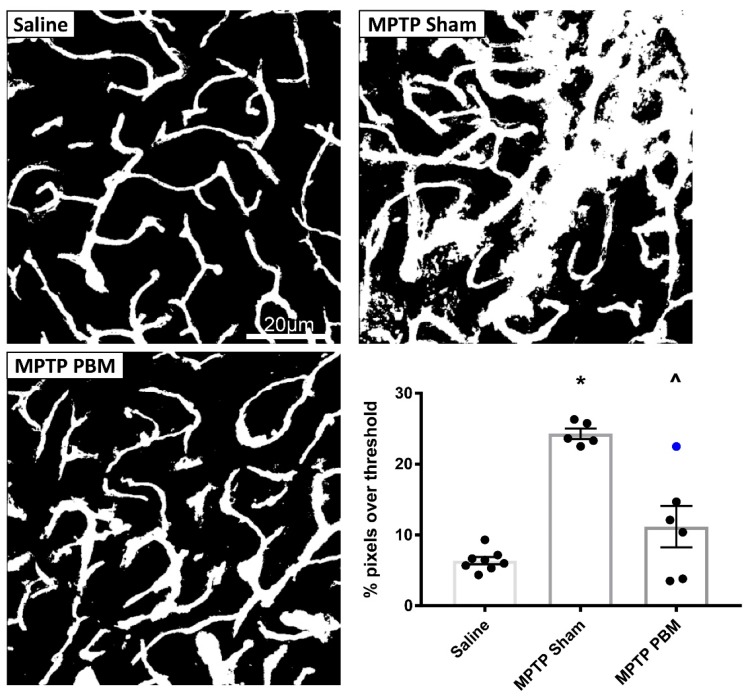
Transcranial PBM mitigates MPTP-induced vascular leakage in the CPu. Top and bottom left panels: Representative thresholded images of FITC-LA labelling in the CPu of mice injected with MPTP and exposed to daily PBM treatment (MPTP PBM) or sham treatment (MPTP Sham), as well as saline-injected controls (Saline). Scale bar in the top left image applies to all images. Bottom right panel: Quantification of the percentage of pixels above threshold, as determined from thresholded FITC-LA images. Data are presented as mean ± SEM (*n* = 8 for saline, *n* = 5 for MPTP Sham, *n* = 6 for MPTP PBM). * *p* < 0.0001 vs. Saline. ^ *p* = 0.0003 vs. MPTP Sham. Blue dot represents the unshaved mouse.

Representative thresholded images of the CPu of mice from each experimental group are shown in Figure 6. Qualitative inspection of images from each animal revealed consistent and extensive leakage in the CPu of all animals from the MPTP-sham group. In contrast, images from mice in the MPTP-PBM group were more variable: some were indistinguishable from saline-injected mice, while others showed evidence of leakage at some vessel branch points, albeit less pronounced than in the MPTP-sham group.

Quantification of FITC-LA labelling in CPu sections is presented in Figure 6. One animal in the MPTP-sham group, which returned a very high value (48%), was excluded as an outlier by ROUT analysis. Relative to saline-injected controls, mice in the MPTP-sham condition showed a nearly 4-fold increase in FITC-LA labelling relative to saline-injected controls. The mean value of the MPTP-PBM group was slightly higher than the saline-injected control mean, and all animals in the MPTP-PBM group (with the exception of the unshaved animal) returned lower vales than all mice in the MPTP-sham group. One-way ANOVA revealed a significant difference in group means (F(2, 17) = 28.77, *p* < 0.0001), with post-hoc Tukey’s multiple comparisons test revealing that the MPTP-sham group was significantly different to saline-injected controls (*p* < 0.0001) and the MPTP-PBM group (*p* = 0.0003). Again, there was no significant difference between saline control and MPTP-PBM groups (*p* = 0.12), suggesting that transcranial PBM mitigates MPTP-induced cerebrovascular leakage to near-control levels.

## 4. Discussion

This project sought to address two primary aims: (i) to determine the time course over which MPTP causes disruption of the brain vasculature, specifically in the nigrostriatal pathway, and (ii) to determine whether transcranial PBM can mitigate MPTP-induced cerebrovascular dysfunction. The findings indicate that MPTP causes substantial disruption of cerebrovascular integrity that appears to be transient, peaking at 7 days post-injection, and that transcranial PBM can effectively mitigate cerebrovascular leakage at the 7 day time point.

### 4.1. Comparison to Previous Studies

Some previous studies that have assessed the effect of MPTP on the brain vasculature have reported damage at 3 days after MPTP exposure [9,10,22]. The present study confirms MPTP-induced damage to the vasculature as assessed by leakage of FITC-LA, peaking at 7 days post-injection, but with no significant leakage apparent at 3 days post-injection. This discrepancy at the 3 day time point is difficult to explain, as there are no obvious differences in methodology between the studies. For example, two studies by Chung and colleagues [9,22], using the same MPTP dosage and injection schedule as the present study, observed widespread FITC-LA leakage in the SNc at 3 days post-MPTP injection (4- to 5-fold increase relative to saline-injected controls). The only methodological differences appear to relate to the image analysis, where the authors sampled smaller fields of view within the SNc rather than performing a tilescan on the whole structure, and the perfusion of 10 mL of FITC-LA solution (in the 2016 study) as opposed to 5 mL in the present study (and most other reported studies). However, it seems unlikely that these factors could account for the observed differences in FITC-LA labelling. Surprisingly, an earlier study by Zhao and colleagues reported FITC-LA leakage in both the SNc and CPu at 3 days post-MPTP administration, despite using half the MPTP concentration (40 mg/kg, administered as 10 mg/kg every hour over 4 h) of the present study; however, no quantification was provided [10].

### 4.2. Potential Mechanisms

#### 4.2.1. MPTP-Induced Cerebrovascular Leakage and BBB Dysfunction

The mechanism by which MPTP disrupts the cerebral vasculature is largely unknown. One prime candidate is through inflammatory processes, since MPTP is known to cause widespread neuroinflammation which in turn could result in BBB dysfunction [23]. MPTP has a strong effect on microglia, activating these cells and causing them to undergo changes in morphology and phenotypic expression (microgliosis), which in turn initiates the release of increased concentrations of reactive oxygen species (ROS) [24,25,26,27]. ROS are known to activate the NF-κB pathway, which is responsible for the phosphorylation of a Kappa inhibitor and, consequently, increased production of pro-inflammatory cytokines [24,28], including interleukin -1, interleukin-6 and tumor necrosis factor [29].

The BBB is particularly sensitive to increased levels of ROS [30]. Astrocytic end foot processes form the outer-most layer of the BBB and have a natural antioxidative capacity [31]. The widespread stress of MPTP-induced inflammation on glial cells and the associated increased levels of ROS would likely exceed the buffering capacity of the astrocyte, leading to astrocytic swelling and BBB disruption [32,33,34]. While tight junction proteins (e.g., occludin, claudin) generally keep endothelial cells cemented together [31], these proteins can be redistributed or have their structure altered as a result of neuroinflammation (i.e., increased levels of ROS and cytokines), leading to compromise of the BBB [35,36]. It will be valuable to explore this possible mechanism in future research, by measuring levels of ROS and cytokines in brain tissue following MPTP insult and assessing whether antioxidants and anti-inflammatory interventions can mitigate MPTP-induced damage to the brain vasculature.

The inflammation hypothesis is also consistent with the observation of partial recovery of cerebrovascular integrity at 14 days post-MPTP injection. Numerous studies have assessed the time course of MPTP-induced microglial activation in both the SNc and CPu, finding that it is an early event that precedes dopaminergic cell death but resolves approximately 7 days after exposure [37]. As MPTP-induced vascular leakage does not appear to be dependent on dopaminergic cell loss—since it is not confined to dopaminergic pathways (see Figure 4B)—it is perhaps not surprising that the time course of vascular dysfunction more closely mirrors that of microglial activation and inflammation than that of nigrostriatal degeneration. Nonetheless, it will be valuable in future studies to extend investigations of the brain vasculature to later time points, in order to confirm that MPTP-induced cerebrovascular compromise is indeed a transient phenomenon.

#### 4.2.2. PBM-Induced Mitigation of Cerebrovascular Dysfunction

While the effects of PBM on the cerebrovasculature have never been assessed, one previous study has shown that PBM mitigates the degeneration and leakage of retinal capillaries in animal models of diabetes [38]. 

The exact mechanisms by which PBM protects the brain vasculature are unknown. There is evidence to suggest that PBM prevents neuroinflammation via many of the secondary effects that result from modulating mitochondrial function [39]. The application of PBM to damaged cells has been found to reduce ROS levels, preventing the activation of the NF-κB pathway [40,41]. Furthermore, PBM has been found to reduce pro-inflammatory cytokine levels in activated inflammatory cells. In dendritic cells, PBM reduced MHC class II and CD86, two cell surface markers of inflammation [42]. PBM has also been found to reduce levels of the pro-inflammatory cytokine tumor necrosis factor α [43,44], and in a mouse model of traumatic brain injury, PBM reduced the quantity of activated microglia [45].

Although there is no direct evidence of PBM acting on the inflammation associated with MPTP intoxication, the initial phases of the inflammatory response are similar across most cells types [46], so the observation of PBM-mediated reductions in inflammation in other tissues is likely to be relevant to the brain. Future studies should confirm this by exploring the effects of PBM on neuroinflammation caused by MPTP; for example, by measuring brain cytokine and chemokine levels and brain glial and immune cell populations.

### 4.3. Strengths and Limitations of the Study

One major strength of this study was that PBM was administered as a post-conditioning intervention, commencing only after MPTP had been injected and fully metabolized. This is in contrast to most previous PBM studies using MPTP mice, where PBM treatment has been applied as a pre-conditioning (i.e., before insult) or per-conditioning (concurrent with insult) protocol [12,13,18,19,47]. Applying PBM as a post-conditioning protocol is more consistent with the clinical reality, where therapeutic interventions generally only commence once damage is apparent and disease is diagnosed.

The use of FITC-LA perfusion as an indicator of vascular dysfunction was preferenced over albumin-binding dyes such as Evans Blue, since unbound Evans Blue has been shown to diffuse across an intact BBB, leading to false positive results [48]. However, one limitation of using FITC-LA is that fluorescent signal is retained within the vessel lumen as well as at sites of leakage, precluding the quantification of leakage specifically. One option that was considered was to follow FITC-LA perfusion with a saline flush, so that only FITC-LA that enters the neuropil is retained in the tissue sections. However, a previous study found that this approach tended to wash away FITC-LA in the neuropil, and that residual FITC-LA was occasionally found in vessels [49]. An alternative approach may be to co-label FITC-LA-perfused sections with an endothelial cell marker, in order to enable the clear delineation of vessel boundaries and the specific visualization of FITC-LA that has leaked beyond these boundaries. However, we observed leaching of FITC-LA from the sections during the incubation steps required for antibody labelling; in future studies, this leaching might be preventable by perfusing a FITC-LA gel that solidifies at a certain temperature [50]. It will also be valuable in future studies to incorporate other measures of BBB dysfunction (e.g., Western immunoblotting or immunohistochemistry of BBB markers such as occludin, claudins or PECAM1) and identify possible drivers of this dysfunction (e.g., changes in glial cell morphology, ROS and proinflammatory molecules such as chemokines and cytokines).

### 4.4. Implications and Future Directions

The findings of the present study have a number of important implications for both the value of MPTP mice as a model of Parkinson’s disease and the potential of PBM to treat a range of different neurological conditions.

#### 4.4.1. Is the MPTP Mouse a Useful Model for Parkinson’s Disease Research?

Exposing animals to the neurotoxin MPTP is the most widely used approach for modelling the nigrostriatal degeneration of PD. The present study characterizes an additional damaging effect of MPTP on brain structure: the apparent disruption of the BBB, leading to the diffusion of molecules as large as 70 kDa between the bloodstream and the brain interstitial fluid.

It is unclear whether the same level of BBB dysfunction and cerebrovascular leakage also occurs in PD patients. Current evidence for cerebrovascular dysfunction in the PD brain is limited to four studies [51,52,53,54], one of which suggests that BBB damage only occurs at the later stages of disease [51]. If cerebrovascular dysfunction resulting from MPTP intoxication is far more extensive than in human patients, can MPTP models still be considered relevant for PD research, particularly in the context of evaluating potential drug therapies?

In pharmacological settings, the BBB is often a major obstacle to delivering drug treatments to the CNS. In order to passively diffuse across an intact BBB, drugs have to be highly lipophilic [55]. Many of the PD treatments available today are limited by their biodynamics and usually have to be converted into their active compounds once they cross the BBB.

One example of an emerging PD treatment, the iron chelator deferiprone, was trialed in an MPTP mouse model in 2014 [56], where it produced significant mitigation of dopaminergic cell death in the SNc, increased dopamine levels throughout the brain, reduced iron accumulation and reduced oxidative stress. However, this treatment yielded underwhelming results in phase 2 clinical trials, producing a small decrease in SNc iron levels in only 3 out of 22 patients [57]. A study of deferiprone in another neurotoxin model of PD, using 6-hydroxydopamine, showed similar protective effects to those in the MPTP model; however, since 6-hydroxydopamine is known to disrupt the BBB, it was proposed by the authors that drug uptake might be enhanced due to cerebrovascular dysfunction [58]. Thus, the mismatch between BBB permeability in toxin-induced PD models and PD patients might explain the failure of certain clinical drug trials, and the deferiprone example should serve as a cautionary tale. Based on the findings of the present study, it is advisable that any drug treatment showing success in MPTP models be trialed in other non-neurotoxin-based PD models (e.g., transgenic mice) before human testing.

#### 4.4.2. Might PBM be an Effective Treatment for Other Brain Diseases?

The present study is the first to investigate the effects of PBM in mitigating leakage of the brain vasculature. Although this was assessed only in an MPTP model, the broad-based efficacy of PBM in protecting against a number of other pathologies [59,60] suggests that PBM might be effective in mitigating vascular dysfunction in other disease contexts. Vascular dysfunction has been well documented in a range of other neurological diseases, including Alzheimer’s disease (AD), multiple sclerosis, Huntington’s disease and stroke [27,61,62,63].

As just one example, dysregulated cerebral blood flow has been identified in the early phases of AD [64]. This includes the observation of microhemorrhages from cerebral capillaries, which result in the accumulation of toxic blood-derived compounds in the neuropil as well as faulty clearance of the important AD peptide amyloid-β (Aβ) and the accumulation of Aβ oligomers [63,65,66]. As a response to disrupted blood perfusion and rising Aβ levels, tau protein accumulates and further obstructs the vessels, increasing the damage [67]. There is evidence from transgenic mouse models of AD and one small clinical trial that PBM can mitigate the neuropathology and cognitive deficits of AD [68,69,70,71], yet none of these studies have investigated whether this is due to improvements in cerebrovascular function. Nonetheless, the strong protective effects of PBM on the brain vasculature observed in the present study suggest that PBM might be useful as a treatment option in a range of neurological diseases that involve vascular dysfunction.

## 5. Conclusions

In summary, we have characterized the timeline of MPTP-induced cerebrovasculature dysfunction, providing evidence that vascular damage is transient, peaking at around 7 days post-injection. Further, we have, for the first time, demonstrated that transcranial PBM is effective in mitigating MPTP-induced damage to the brain vasculature. These findings open up a number of avenues for future studies, including research into the mechanisms by which MPTP disrupts the brain vasculature, whether BBB dysfunction is a common feature among PD patients, and the translatability of PBM over the range of brain conditions that involve BBB dysfunction.

## References

[1] Langston J.W. (2017). The MPTP Story. J. Parkinsons Dis..

[2] Liss B., Haeckel O., Wildmann J., Miki T., Seino S., Roeper J. (2005). K-ATP channels promote the differential degeneration of dopaminergic midbrain neurons. Nat. Neurosci..

[3] Meredith G.E., Kang U.J. (2006). Behavioral models of Parkinson’s disease in rodents: A new look at an old problem. Mov. Disord..

[4] Meredith G.E., Rademacher D.J. (2011). MPTP Mouse Models of Parkinson’s Disease: An Update. J. Park. Dis..

[5] Sedelis M., Hofele K., Auburger G.W., Morgan S., Huston J.P., Schwarting R.K.W. (2000). MPTP Susceptibility in the Mouse: Behavioral, Neurochemical, and Histological Analysis of Gender and Strain Differences. Behav. Genet..

[6] Chao Y.X., He B.P., Tay S.S.W. (2009). Mesenchymal stem cell transplantation attenuates blood brain barrier damage and neuroinflammation and protects dopaminergic neurons against MPTP toxicity in the substantia nigra in a model of Parkinson’s disease. J. Neuroimmunol..

[7] Chen X., Lan X., Roche I., Liu R., Geiger J.D. (2008). Caffeine protects against MPTP-induced blood-brain barrier dysfunction in mouse striatum. J. Neurochem..

[8] Choi J.H., Jang M., Oh S., Nah S.-Y., Cho I.-H. (2018). Multi-Target Protective Effects of Gintonin in 1-Methyl-4-phenyl-1,2,3,6-tetrahydropyridine-Mediated Model of Parkinson’s Disease via Lysophosphatidic Acid Receptors. Front. Pharmacol..

[9] Chung Y.C., Shin W.-H., Baek J.Y., Cho E.J., Baik H.H., Kim S.R., Won S.-Y., Jin B.K. (2016). CB2 receptor activation prevents glial-derived neurotoxic mediator production, BBB leakage and peripheral immune cell infiltration and rescues dopamine neurons in the MPTP model of Parkinson’s disease. Exp. Mol. Med..

[10] Zhao C., Ling Z., Newman M.B., Bhatia A., Carvey P.M. (2007). TNF-alpha knockout and minocycline treatment attenuates blood-brain barrier leakage in MPTP-treated mice. Neurobiol. Dis..

[11] El Massri N., Johnstone D.M., Peoples C.L., Moro C., Reinhart F., Torres N., Stone J., Benabid A.L., Mitrofanis J. (2016). The effect of different doses of near infrared light on dopaminergic cell survival and gliosis in MPTP-treated mice. Int J. Neurosci.

[12] Johnstone D., El Massri N., Moro C., Spana S., Wang X., Torres N., Chabrol C., De Jaeger X., Reinhart F., Purushothuman S. (2014). Indirect application of near infrared light induces neuroprotection in a mouse model of parkinsonism—An abscopal neuroprotective effect. Neuroscience.

[13] Moro C., Torres N., El Massri N., Ratel D., Johnstone D.M., Stone J., Mitrofanis J., Benabid A.-L. (2013). Photobiomodulation preserves behaviour and midbrain dopaminergic cells from MPTP toxicity: Evidence from two mouse strains. BMC Neurosci..

[14] Peoples C., Spana S., Ashkan K., Benabid A.-L., Stone J., Baker G.E., Mitrofanis J. (2012). Photobiomodulation enhances nigral dopaminergic cell survival in a chronic MPTP mouse model of Parkinson’s disease. Park. Relat. Disord..

[15] Reinhart F., El Massri N., Johnstone D.M., Stone J., Mitrofanis J., Benabid A.-L., Moro C. (2016). Near-infrared light (670 nm) reduces MPTP-induced parkinsonism within a broad therapeutic time window. Exp. Brain Res..

[16] Reinhart F., El Massri N., Darlot F., Torres N., Johnstone D.M., Chabrol C., Costecalde T., Stone J., Mitrofanis J., Benabid A.-L. (2015). 810nm near-infrared light offers neuroprotection and improves locomotor activity in MPTP-treated mice. Neurosci. Res..

[17] Reinhart F., El Massri N., Torres N., Chabrol C., Molet J., Johnstone D.M., Stone J., Benabid A.-L., Mitrofanis J., Moro C. (2017). The behavioural and neuroprotective outcomes when 670 nm and 810 nm near infrared light are applied together in MPTP-treated mice. Neurosci. Res..

[18] Shaw V.E., Spana S., Ashkan K., Stone J., Baker G.E., Mitrofanis J., Benabid A.-L., Benabid A. (2010). Neuroprotection of midbrain dopaminergic cells in MPTP-treated mice after near-infrared light treatment. J. Comp. Neurol..

[19] Ganeshan V., Skladnev N.V., Kim J.Y., Mitrofanis J., Stone J., Johnstone D.M. (2019). Pre-conditioning with Remote Photobiomodulation Modulates the Brain Transcriptome and Protects Against MPTP Insult in Mice. Neuroscience.

[20] Kim B., Mitrofanis J., Stone J., Johnstone D.M. (2018). Remote tissue conditioning is neuroprotective against MPTP insult in mice. IBRO Rep..

[21] Jackson-Lewis V., Przedborski S. (2007). Protocol for the MPTP mouse model of Parkinson’s disease. Nat. Protoc..

[22] Chung Y.C., Kim Y.-S., Bok E., Yune T.Y., Maeng S., Jin B.K. (2013). MMP-3 Contributes to Nigrostriatal Dopaminergic Neuronal Loss, BBB Damage, and Neuroinflammation in an MPTP Mouse Model of Parkinson’s Disease. Mediat. Inflamm..

[23] Mendes M.O., Rosa A.I., Carvalho A.N., Nunes M.J., Dionísio P., Rodrigues E., Costa D., Duarte-Silva S., Maciel P., Rodrigues C.M.P. (2019). Neurotoxic effects of MPTP on mouse cerebral cortex: Modulation of neuroinflammation as a neuroprotective strategy. Mol. Cell. Neurosci..

[24] Beal M.F. (2003). Mitochondria, oxidative damage, and inflammation in Parkinson’s disease. Ann. N. Y. Acad. Sci..

[25] Kraft A.D., Harry G.J. (2011). Features of Microglia and Neuroinflammation Relevant to Environmental Exposure and Neurotoxicity. Int. J. Environ. Res. Public Heal..

[26] Vroon A., Drukarch B., Bol J.G., Cras P., Brevé J.J., Allan S.M., Relton J.K., Hoogland P.V., Van Dam A.-M. (2007). Neuroinflammation in Parkinson’s patients and MPTP-treated mice is not restricted to the nigrostriatal system: Microgliosis and differential expression of interleukin-1 receptors in the olfactory bulb. Exp. Gerontol..

[27] Zlokovic B.V. (2008). The Blood-Brain Barrier in Health and Chronic Neurodegenerative Disorders. Neuron.

[28] A O’Neill L., Kaltschmidt C. (1997). NF-kappa B: A crucial transcription factor for glial and neuronal cell function. Trends Neurosci..

[29] Becher B., Spath S., Goverman J. (2017). Cytokine networks in neuroinflammation. Nat. Rev. Immunol.

[30] Peterson L.J., Flood P.M. (2012). Oxidative Stress and Microglial Cells in Parkinson’s Disease. Mediat. Inflamm..

[31] Abbott N.J., Patabendige A.A., Dolman D.E., Yusof S.R., Begley D.J. (2010). Structure and function of the blood–brain barrier. Neurobiol. Dis..

[32] Abbott N.J., Rönnbäck L., Hansson E., R L. (2006). Astrocyte–endothelial interactions at the blood–brain barrier. Nat. Rev. Neurosci..

[33] Cabezas R., Avila M., Gonzalez J., El-Bacha R.S., Baez E., Garcia-Segura L.M., Jurado Coronel J.C., Capani F., Cardona-Gomez G.P., Barreto G.E. (2014). Astrocytic modulation of blood brain barrier: Perspectives on Parkinson’s disease. Front. Cell Neurosci.

[34] Chen Z.-L., Indyk J.A., Bugge T.H., Kombrinck K.W., Degen J.L., Strickland S. (1999). Neuronal Death and Blood–Brain Barrier Breakdown after Excitotoxic Injury Are Independent Processes. J. Neurosci..

[35] Capaldo C.T., Farkas A.E., Hilgarth R.S., Krug S.M., Wolf M.F., Benedik J.K., Fromm M., Koval M., Parkos C., Nusrat A. (2014). Proinflammatory cytokine-induced tight junction remodeling through dynamic self-assembly of claudins. Mol. Boil. Cell.

[36] Greene C., Campbell M. (2016). Tight junction modulation of the blood brain barrier: CNS delivery of small molecules. Tissue Barriers.

[37] Huang D., Xu J., Wang J., Tong J., Bai X., Li H., Wang Z., Huang Y., Wu Y., Yu M. (2017). Dynamic Changes in the Nigrostriatal Pathway in the MPTP Mouse Model of Parkinson’s Disease. Park. Dis..

[38] Cheng Y., Du Y., Liu H., Tang J., Veenstra A., Kern T.S. (2018). Photobiomodulation Inhibits Long-term Structural and Functional Lesions of Diabetic Retinopathy. Diabetes.

[39] Hamblin M.R. (2017). Mechanisms and applications of the anti-inflammatory effects of photobiomodulation. AIMS Biophys..

[40] De Marchi T., Leal Junior E.C., Bortoli C., Tomazoni S.S., Lopes-Martins R.A., Salvador M. (2012). Low-level laser therapy (LLLT) in human progressive-intensity running: Effects on exercise performance, skeletal muscle status, and oxidative stress. Lasers Med. Sci..

[41] Tatmatsu-Rocha J.C., Ferraresi C., Hamblin M.R., Damasceno F.M., Nascimento N.R.F.D., Driusso P., Parizotto N.A. (2016). Low-Level Laser Therapy (904nm) Can Increase Collagen and Reduce Oxidative and Nitrosative Stress in Diabetic Wounded Mouse Skin. J. Photochem. Photobiol. B: Boil..

[42] Chen A.C.-H., Huang Y.-Y., Sharma S.K., Hamblin M.R. (2011). Effects of 810-nm Laser on Murine Bone-Marrow-Derived Dendritic Cells. Photomed. Laser Surg..

[43] Hwang M.H., Shin J.H., Kim K.S., Yoo C.M., Jo G.E., Kim J.H., Choi H. (2015). Low Level Light Therapy Modulates Inflammatory Mediators Secreted by Human Annulus Fibrosus Cells during Intervertebral Disc Degeneration In Vitro. Photochem. Photobiol..

[44] Yamaura M., Yao M., Yaroslavsky I., Cohen R., Smotrich M., Kochevar I.E. (2009). Low level light effects on inflammatory cytokine production by rheumatoid arthritis synoviocytes. Lasers Surg. Med..

[45] Khuman J., Zhang J., Park J., Carroll J.D., Donahue C., Whalen M.J. (2012). Low-Level Laser Light Therapy Improves Cognitive Deficits and Inhibits Microglial Activation after Controlled Cortical Impact in Mice. J. Neurotrauma.

[46] Rock K.L., Kono H. (2008). The inflammatory response to cell death. Annu. Rev. Pathol. Mech. Dis..

[47] Whelan H. (2008). Harnessing the cell’s own ability to repair and prevent neurodegenerative disease. SPIE Newsroom.

[48] Saunders N.R., Dziegielewska K.M., Mollgard K., Habgood M.D. (2015). Markers for blood-brain barrier integrity: How appropriate is Evans blue in the twenty-first century and what are the alternatives?. Front. Neurosci..

[49] Natarajan R., Northrop N., Yamamoto B. (2017). Fluorescein Isothiocyanate (FITC)-Dextran Extravasation as a Measure of Blood-Brain Barrier Permeability.

[50] Blinder P., Tsai P.S., Kaufhold J.P., Knutsen P.M., Suhl H., Kleinfeld D. (2013). The cortical angiome: An interconnected vascular network with noncolumnar patterns of blood flow. Nat. Neurosci..

[51] Bartels A.L., Willemsen A.T.M., Kortekaas R., De Jong B.M., De Vries R., De Klerk O., Van Oostrom J.C.H., Portman A., Leenders K.L. (2008). Decreased blood–brain barrier P-glycoprotein function in the progression of Parkinson’s disease, PSP and MSA. J. Neural Transm..

[52] Desai Bradaric B., Patel A., Schneider J.A., Carvey P.M., Hendey B. (2012). Evidence for angiogenesis in Parkinson’s disease, incidental Lewy body disease, and progressive supranuclear palsy. J. Neural Transm. (Vienna).

[53] Gray M.T., Woulfe J.M. (2015). Striatal blood–brain barrier permeability in Parkinson’s disease. Br. J. Pharmacol..

[54] Kortekaas R., Leenders K.L., Van Oostrom J.C.H., Vaalburg W., Bart J., Willemsen A.T.M., Hendrikse N.H. (2005). Blood-brain barrier dysfunction in parkinsonian midbrain in vivo. Ann. Neurol..

[55] A Banks W. (2009). Characteristics of compounds that cross the blood-brain barrier. BMC Neurol.

[56] Devos D., Moreau C., Devedjian J.C., Kluza J., Petrault M., Laloux C., Jonneaux A., Ryckewaert G., Garçon G., Rouaix N. (2014). Targeting Chelatable Iron as a Therapeutic Modality in Parkinson’s Disease. Antioxidants Redox Signal..

[57] Martin-Bastida A., Ward R.J., Newbould R., Piccini P., Sharp D., Kabba C., Patel M.C., Spino M., Connelly J., Tricta F. (2017). Brain iron chelation by deferiprone in a phase 2 randomised double-blinded placebo controlled clinical trial in Parkinson’s disease. Sci. Rep..

[58] Dexter D.T., Statton S.A., Whitmore C., Freinbichler W., Weinberger P., Tipton K.F., Della Corte L., Ward R.J., Crichton R.R. (2011). Clinically available iron chelators induce neuroprotection in the 6-OHDA model of Parkinson’s disease after peripheral administration. J. Neural Transm. (Vienna).

[59] Hamblin M.R. (2016). Shining light on the head: Photobiomodulation for brain disorders. BBA Clin..

[60] Johnstone D.M., Moro C., Stone J., Benabid A.-L., Mitrofanis J. (2016). Turning On Lights to Stop Neurodegeneration: The Potential of Near Infrared Light Therapy in Alzheimer’s and Parkinson’s Disease. Front. Neurosci..

[61] Minagar A., Alexander J.S. (2003). Blood-brain barrier disruption in multiple sclerosis. Mult. Scler. J..

[62] Rosenberg G. (2014). Blood-Brain Barrier Permeability in Aging and Alzheimer’s Disease. J. Prev. Alzheimer’s Dis..

[63] Sweeney M.D., Sagare A.P., Zlokovic B.V. (2018). Blood–brain barrier breakdown in Alzheimer disease and other neurodegenerative disorders. Nat. Rev. Neurol..

[64] Zlokovic B.V. (2011). Neurovascular pathways to neurodegeneration in Alzheimer’s disease and other disorders. Nat. Rev. Neurosci..

[65] Cullen K.M., Kócsi Z., Stone J. (2005). Pericapillary Haem-Rich Deposits: Evidence for Microhaemorrhages in Aging Human Cerebral Cortex. Br. J. Pharmacol..

[66] Stone J., Johnstone D.M., Mitrofanis J., O’Rourke M. (2015). The mechanical cause of age-related dementia (Alzheimer’s disease): The brain is destroyed by the pulse. J. Alzheimer’s Dis..

[67] Cai Z., Qiao P.-F., Wan C.-Q., Cai M., Zhou N.-K., Li Q. (2018). Role of Blood-Brain Barrier in Alzheimer’s Disease. J. Alzheimer’s Dis..

[68] De Taboada L., Yu J., El-Amouri S., Gattoni-Celli S., Richieri S., McCarthy T., Streeter J., Kindy M.S. (2011). Transcranial laser therapy attenuates amyloid-beta peptide neuropathology in amyloid-beta protein precursor transgenic mice. J. Alzheimers Dis..

[69] Grillo S.L., Duggett N.A., Ennaceur A., Chazot P.L. (2013). Non-invasive infra-red therapy (1072 nm) reduces beta-amyloid protein levels in the brain of an Alzheimer’s disease mouse model, TASTPM. J. Photochem. Photobiol. B.

[70] Purushothuman S., Johnstone D.M., Nandasena C., Mitrofanis J., Stone J. (2014). Photobiomodulation with near infrared light mitigates Alzheimer’s disease-related pathology in cerebral cortex—Evidence from two transgenic mouse models. Alzheimer’s Res. Ther..

[71] Saltmarche A.E., Naeser M.A., Ho K.F., Hamblin M.R., Lim L. (2017). Significant Improvement in Cognition in Mild to Moderately Severe Dementia Cases Treated with Transcranial Plus Intranasal Photobiomodulation: Case Series Report. Photomed. Laser Surg..

